# Reliability of Inertial Measurement Unit-Based Spatiotemporal and Kinetic Variables in Endurance Runners during Treadmill Running

**DOI:** 10.5114/jhk/195895

**Published:** 2025-05-12

**Authors:** Unai Miqueleiz, Roberto Aguado-Jimenez, Pablo Lecumberri, Ibai Garcia-Tabar, Esteban M. Gorostiaga

**Affiliations:** 1 Department of Health Sciences, Public University of Navarre, Pamplona, Spain.; 2 Studies, Research and Sports Medicine Centre (CEIMD), Government of Navarre, Pamplona, Spain.; 3 Department of Mathematics, Public University of Navarre, Pamplona, Spain.; 4 Society, Sports and Physical Exercise Research Group (GIKAFIT), Department of Physical Education and Sport, Faculty of Education and Sport, University of the Basque Country (UPV/EHU), Vitoria-Gasteiz, Spain.; 5 Physical Activity, Exercise, and Health group, Bioaraba Health Research Institute, Vitoria-Gasteiz, Basque Country, Spain.

**Keywords:** biomechanics, inertial sensor, wearable devices, aerobic capacity, accelerometer

## Abstract

Wearable technology for running analysis is growing in both sports science research and applied fields. This study examined the reliability of some spatiotemporal and kinetic variables estimated from an inertial measurement unit (IMU) fastened over the lumbar spine. Eighteen recreational endurance runners performed two maximal incremental treadmill running tests during a 7–10 day period under standard conditions. Contact time (CT), stride time (ST), stride length (SL), stride frequency (SF), as well as anteroposterior (AP) impulses and vertical (VT) peak brake data were analysed at 9, 15 and 21 km•h^−1^. Test-retest reliability was measured as the intraclass correlation coefficient (ICC), the coefficient of variation (CV) and minimal detectable change (MDC). No significant differences between tests were observed (p > 0.05; effect size (ES) < 0.28; trivial to small). Reliability increased from 9 to 21 km•h^−1^ (ICC from 0.88 to 0.93; ES = 1.0; moderate) and was higher in spatiotemporal (CV < 2.3%) than kinetic variables (CV < 6.8%). This study adds novel data regarding the reliability of the MTw IMU. The results reported in this study enable researchers to determine whether the changes in IMU-derived data are outside of the measurement error following training and rehabilitation settings.

## Introduction

Running biomechanics can examine how the body moves and the effects that repeated ground contact has on the body. These analyses have historically employed force plates and high-speed cameras to analyse the movements of the body. However, these technologies are very expensive, time-consuming, and mostly limited to laboratory settings (Kluitenberg et al., 2012). In the last decade, inertial measurement units (IMUs) have been implemented for the study of human locomotion (Chew et al., 2018; Gouttebarge et al., 2015). The advantage of using IMUs is that they provide on-field and laboratory analysis of temporal events in a wearable and low-cost manner (Van Hooren et al., 2020). IMUs, such as the MTw IMU (Xsens technology), are gaining special interest due to their low-cost and general availability. Coaches, physiotherapists, and other exercise-related practitioners have begun using this kind of devices to analyse human locomotion for clinical rehabilitation (Moon et al., 2017; Tura et al., 2010) and sport performance purposes (Setuain et al., 2016).

There are some spatiotemporal and kinetic IMU-based variables that could be measured and estimated, respectively, and then analysed with the corresponding software. Contact time (CT) and stride frequency (SF) are some of the spatiotemporal variables analysed in previous research (Gindre et al., 2015). Regarding kinetic variables, several studies have examined the vertical ground reaction forces (vGRFs) and shock attenuation through the body during running (Buchheit et al., 2015; Mercer et al., 2002; Reenalda et al., 2019). However, there is little research regarding the anteroposterior braking and propulsion impulses with IMUs, despite they are the main contributors to the change in horizontal velocity of the centre of mass (CoM) during running (Hunter et al., 2005; Morin et al., 2015).

The applicability of these IMU variables is contingent on measurement error. Knowledge of devices’ reliability allows assessment of whether the variables measures are consistent in the same population on two separate occasions. Such evaluations also enable researchers to interpret whether practically meaningful inferences can be drawn from changes in the data (i.e., whether the changes are outside of the error of measurement). Previous studies analysed the reliability of spatiotemporal variables in recreational runners during both overground (Gindre et al., 2015; Gouttebarge et al., 2015) and treadmill running (Garcia-Pinillos et al., 2019, 2021) at speeds between 8 and 21 km∙h^−1^. Besides, vGRF was studied in healthy adults (Eggers et al., 2018) and professional triathletes (Raper et al., 2018) during overground running at 12 and 18 km∙h^−1^, respectively. However, there is no research concerning the reliability of the AP impulse-based variables. To the best of our knowledge, there is no literature regarding the reliability of these IMU variables during treadmill running at different speeds in endurance runners. Therefore, the aim of the present study was to evaluate, through a test-retest design, the reliability of the IMU-based variables in endurance runners while running on a treadmill at different incremental speeds. This would provide information concerning the minimal change that ensures a real difference after an intervention period.

## Methods

### Participants

Thirteen male (38.2 ± 11.6 years; body mass: 69.2 ± 6.6 kg; body height: 177 ± 5 cm) and five female (35.4 ± 8.8 years; body mass: 55.0 ± 4.5 kg; body height: 165 ± 4 cm) recreational endurance runners, familiarized with treadmill running, volunteered. Participants were required to run > 2 days per week over the last year. This level corresponds to an averaged 10.000-m running time of 45’14”. Exclusion criteria listed any musculoskeletal injury in the previous 3 months before the beginning of the study. Runners were required to complete the test-retest within 7–10 days. Participants arrived at each testing session free of alcohol and caffeine intake 24 hours before the tests. Participants were asked to wear their usual running shoes during both tests. Laboratory conditions were within standard conditions (19.8 ± 0.6°C and 27 ± 2.7% humidity). This study was approved by the ethics committee of the Public University of Navarre (approval code: PI/012-20; approval date: 01 April 2016) according to the Declaration of Helsinki. Prior to any testing, the objectives, benefits, and possible risks derived from the study were explained and informed consent was signed.

### Testing Procedures

**Figure 1:**
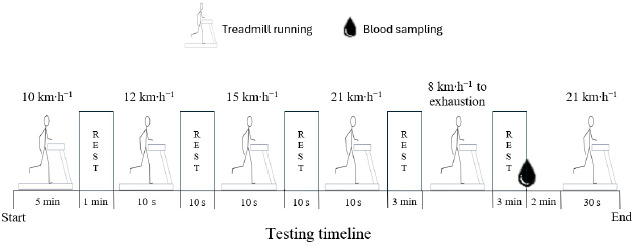
Overview of the experimental protocol.

After participants’ body mass and height were collected, one IMU (MTw, 3DOF Human Orientation Tracker, Xsens Technologies B.V. Enschede, the Netherlands) was strapped over the lumbar spine at the L4–L5 level which represents the centre of mass acceleration (Setuain et al., 2016). Each testing session consisted of running on a treadmill-ergometer (ERG-ELEK-EG4, ISSA Engineers, Vitoria, Spain), of which speed was electronically verified by its photocell timer. Each session (Figure 1) started with a warm-up consisting of 5 min of running at 10 km∙h^−1^ followed by three sets of 10 s at speeds of 12, 15 and 21 km∙h^−1^. After 3 min of rest, participants performed a maximal incremental continuous running test to exhaustion. The treadmill slope was fixed at 1% of elevation. Initial speed was 8 km∙h^−1^ and was incremented by 1 km∙h^−1^ every min (Scrimgeour et al., 1986). After 3 min of the cessation of the test, a capillary whole-blood sample for blood lactate concentration (BLC) was obtained. Finally, 5 min after the end of the incremental test, participants stepped directly onto the moving treadmill (1% slope) and run 30 s at 21 km∙h^−1^.

### Data Processing and Analysis

A fusion algorithm combined the output from accelerometers, gyroscopes and magnetometers to compute the orientation of the IMU in 3D space. Acceleration and orientation signals were recorded (Movalsys, Navarre, Spain), and the automated IMU data analysis procedures were implemented with MatLab R2016a (MathWorks Inc; Natick, MA, USA). The sampling rate was set at 120 Hz and the acceleration signals were low-pass filtered with a zero-lag Butterworth filter (25 Hz cut-off frequency) to reduce high-frequency noise and smooth waveforms (Winter et al., 2018). Twenty seconds of recording at each speed of the incremental test were analysed, beginning at the 20^th^ second of each 60-s running speed studied, and average values (for every participant per speed condition) were calculated for further analysis. At 21 km∙h^−1^, recording started from the 10^th^ second of each 30-s set. Twenty seconds were considered sufficient to ensure the analysis of a minimum of 10 complete strides in all subjects and speeds (Mercer et al., 2002). Time-window analysis at each speed was preferred over a fixed number of strides to avoid intentional selection and counteract the decrement in the number of data points with increasing speed.

**Figure 2:**
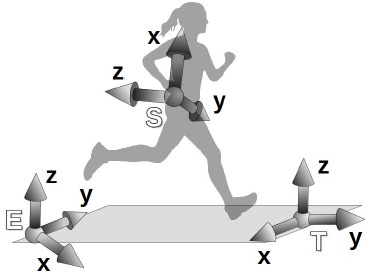
New treadmill-fixed reference frame T representation.

The IMU sensor fusion algorithm computed the orientation of the sensor-fixed reference frame S with respect to an Earth-fixed reference frame E, which was defined with the Z-axis lying on the vertical pointing up and the X-axis pointing to the magnetic North. However, frame E was not related to the treadmill. Since the sensor frame was fixed to the runner’s back, its orientation allowed tracking the runner’s heading (Tan et al., 2008). An additional treadmill-fixed reference frame T was defined by rotating frame E so that the Y-axis pointed to the average heading direction (Figure 2). Furthermore, the runner’s heading in the T reference frame, that is, the projection of a vector along the negative z-axis of the S reference frame onto the x-y plane of the T reference frame, was used to determine the leg of each contact.

### Measures

#### Biomechanical IMU Measures of Endurance Running Performance

Individual data were evaluated to obtain a set of IMU descriptive variables. These included spatiotemporal and kinetic variables identified as representative descriptors of running.

#### Spatiotemporal Variables

**Figure 3:**
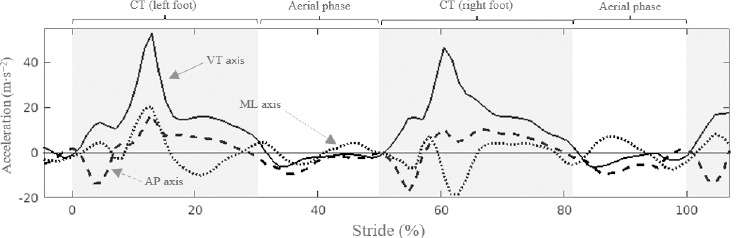
Example of running task phases from an acceleration by stride (%) curve analysis description. Horizontal line corresponds to the 0-acceleration value (m∙s^−2^). Sold line corresponds to the vertical (VT) axis. Dashed line corresponds to the anteroposterior (AP) axis. Dotted line corresponds to the mediolateral (ML) axis. The shading area indicates contact intervals for the left and right legs. CT = Contact time.

Figure 3 shows an example of the acceleration-stride pattern in the VT, ML and AP directions of the track-fixed reference system T.

The beginning of CT (s) was set at the time of the last positive peak in the filtered AP acceleration signal before the pronounced braking (negative AP acceleration peak) that occurred when the foot touched the ground. The end of the contact was established at the first point where AP acceleration was negative again and VT acceleration was below g/2. Stride time (ST, s) was calculated as the time difference between the beginning of two consecutive contacts of the same foot. Stride length (SL, m) resulted from the product of ST (s) and treadmill speed (m∙s^−1^), whereas SF (strides∙min^−1^) was defined as ST^−1^.

The runner’s heading at first contact allowed to discriminate between legs. When the right leg made contact with the floor, the runner’s torso was rotated towards the right and the angle between the z-axis of the sensor-based reference system and the y-axis of the track-based reference system took negative values. Conversely, when the left leg made contact, this angle took positive values.

#### Kinetic Variables

**Figure 4:**
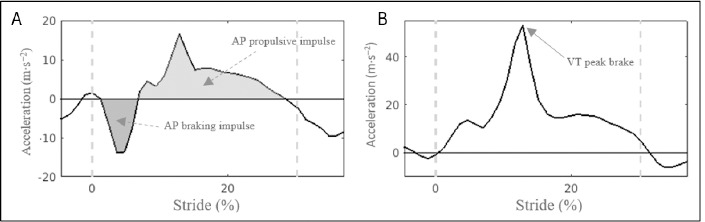
A: AnteroPosterior (AP) impulses. B: Vertical (VT) peak. Dashed lines delineate the contact and aerial phases.

Figure 4A plots the AP acceleration-stride pattern during the contact and the beginning of the aerial phase from a stride in one representative subject. The contact phase was novelty divided into braking and propulsion sub-phases according to negative and positive horizontal accelerations, respectively. AP braking and propulsive impulses (m∙s^−1^) were defined as the area under the acceleration curve during the braking and propulsive phases, respectively, in each contact phase. These impulses represented the change of velocity in the braking and propulsion phases. It is worth noting that during the aerial phase of the stride, there was a negative acceleration pattern. To the best of our knowledge, this is the first study in analysing the role of accelerometry in the AP impulse-based variables. VT peak brake (m∙s^−2^) was defined as the maximal value in the VT axis (Figure 4B).

### Statistical Analysis

Measures were expressed as mean ± SD. After ensuring the normal distribution of the data set (Shapiro-Wilk and Levene’s tests) and when appropriate sphericity (Mauchly’s test), both the paired *t*-test to detect differences between testing sessions and one-way analysis of variance (ANOVA) with pairwise comparisons (Bonferroni’s adjustment) to detect differences among speeds were performed. The magnitudes of the differences were evaluated by Hedge’s effect size (ES), of which magnitude was interpreted as *trivial* (≤ 0.19), *small* (0.20 to 0.59), *moderate* (0.60 to 1.19), *large* (1.20 to 1.99), or *very large* (≥ 2.0) (Hopkins et al., 2009). Relative reliability analysis was performed to calculate the intraclass correlation coefficient (ICC) (2,1), the two-way mixed-effects model and absolute agreement, with a value less than 0.5 as indicative of poor reliability, between 0.5 and 0.75 as indicative of moderate reliability, between 0.75 and 0.9 as indicative of good reliability and values greater than 0.9 as indicative of excellent reliability (Koo and Li, 2016). ICCs were expressed with the confidence interval (95% CI). Absolute reliability was calculated by the standard error of measurement (SEM) of paired data (SEM = SD_diff_/√2) which was expressed as the percentage of SEM divided by the mean, defined as the coefficient of variation (CV) and classified as trivial (CV ≤ 5%) or small (5% < CV ≤ 10%) (Buchheit et al., 2015). Minimal detectable change 90% (MDC_90_ = SEM*1.65*√2) was also calculated. Means, CV, and MDC were calculated at 9, 15, and 21 km∙h^−1^ since all participants were able to run during the established time at those speeds. The Pearson correlation coefficient (r) was utilized to determine the strength of the correlations between CT values. Significance was set at *p* < 0.05. Linear regressions were used to study the presence of systematic errors between days at the speeds tested (proportional difference between the observed and the true values due to the measuring instrument) and ANOVA was used to examine the presence of random errors (unpredictable difference between the observed and the true values due to the measuring instrument or environmental conditions). The statistical analysis was performed with SPSS version 20.0 (SPSS Inc. Chicago, USA).

## Results

**Figure 5:**
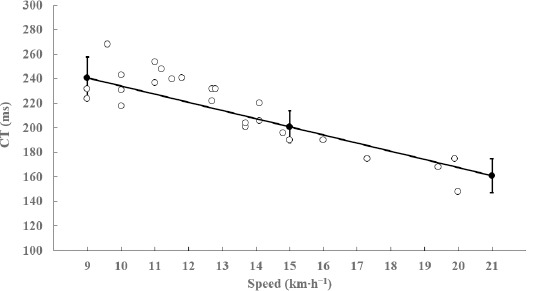
Contact time (CT) comparison between values from this study (mean ± SD; filled circles) and those published with instrumented treadmills (empty circles) in the speed range 9–15–21 km•h^−1^. References: Alcantara et al., 2021; Bredeweg et al., 2013; Giandolini et al., 2013; Girard et al., 2016; Hunter et al., 2019; Kluitenberg et al., 2012; Morin et al., 2012; 2019; Seeley et al., 2020; Weart et al., 2020.

Table 1 shows no differences concerning all measured variables at all three speeds between the first and the second test (*p* > 0.05; ES < 0.26; *trivial to small*). Linear regression between both days for each variable at any speed showed the presence of non-significant systematic errors (R^2^ < 0.07; *p* > 0.05). ANOVA showed the presence of random errors in CT (0.04 ms), ST (0.12 ms), SL (0.002 m), SF (0.001 strides∙min^−1^), the AP braking impulse (0.001 m∙s^−1^), the AP propulsive impulse (0.003 m∙s^−1^), and VT peak brake (0.42 m∙s^−2^). The spatiotemporal variables showed significant differences among the all considered speeds (9, 15 and 21 km∙h^−1^; *p* < 0.01; ES range= 2.5–5.4; *very large*). Figure 5 shows similar IMU-based CT values from 9 to 21 km∙h^−1^ compared with those published with instrumented treadmills (mean difference: 2.4 ms; r = 0.90; *p* < 0.001). Concerning kinetic variables: the AP braking impulse was only different between 9 and 21 km∙h^−1^ (*p* < 0.01; ES = 0.80; *moderate*), the AP propulsive impulse was significantly different among the three speeds (*p* < 0.05; ES range = 0.82–2.6; *moderate to very large*), and VT peak brake was not different between any speed (*p* > 0.05; ES < 0.67; *trivial to moderate*).

**Table 1:** Mean ± SD of the IMU variables analysed at the three selected speeds.

Category	Variable	9 km∙h^−1^		15 km∙h^−1^		21 km∙h^−1^	
		Day 1	Day 2	Day 1	Day 2	Day 1	Day 2
Spatiotemporal	CT (ms)	242 ± 17	240 ± 17	200 ± 13^a^	202 ± 13^a^	161 ± 12^a,b^	161 ± 14^a,b^
	ST (ms)	735 ± 40	734 ± 32	669 ± 41^a^	668 ± 38^a^	583 ± 47^a,b^	586 ± 48^a,b^
	SL (m)	1.84 ± 0.10	1.83 ± 0.08	2.79 ± 0.17^a^	2.78 ± 0.16^a^	3.40 ± 0.27^a,b^	3.42 ± 0.28^a,b^
	SF (strides∙min^−1^)	81.8 ± 4.7	82.0 ± 3.7	90.1 ± 5.8^a^	90.1 ± 5.4^a^	103.6 ± 8.8^a,b^	103.1 ± 9.1^a,b^
Kinetic	AP braking impulse (m∙s^−1^)	−0.28 ± 0.07	−0.28 ± 0.06	−0.34 ± 0.08	−0.32 ± 0.06	−0.35 ± 0.10^a^	−0.34 ± 0.07^a^
	AP prop. impulse (m∙s^−1^)	0.48 ± 0.09	0.46 ± 0.08	0.67 ± 0.11^a^	0.66 ± 0.11^a^	0.79 ± 0.16^a,b^	0.78 ± 0.15^a,b^
	VT peak brake (m∙s^−2^)	41.4 ± 10.6	40.9 ± 11.6	45.6 ± 10.2	45.1 ± 9.3	48.5 ± 10.9	47.5 ± 11.7

ST = Stride Time; SL = Stride Length; SF = Stride Frequency; CT = Contact Time; AP = Antero–Posterior; VT = Vertical. Significant differences: ^a^ Different from 9 km•h^−1^; ^b^ Different from 15 km•h^−1^

Table 2 shows that higher ICC values were observed in the spatiotemporal (0.86–0.98) compared with the kinetic (0.81–0.90) variables (*p* < 0.001; ES = 2.7; *very large*). Eleven out of twelve spatiotemporal ICCs were higher than 0.90 (all the ICCs at 15 and 21 km∙h^−1^), with good to excellent reliability, while in the kinetic variables the ICCs were all higher than 0.80, with good reliability. CVs at 15 and 21 km∙h^−1^ were trivial in the spatiotemporal variables and small in the kinetic variables. Considering the whole set of spatiotemporal variables, ANOVA showed that ICCs significantly increased from 9 to 15–21 km∙h^−1^ (*p* < 0.05; ES = 2.5 and 2.0, *very large*, respectively) and CV remained similar with increasing speed (*p >* 0.05). When considering the whole set of kinetic variables, both ICCs and CVs remained similar with increasing speed (*p >* 0.05).

**Table 2:** Intraclass correlation coefficient (ICC), 95% confidence interval (CI), coefficient of variation (CV) and minimal detectable change (MDC) of the IMU variables analysed at the three selected speeds.

Category	Variable	9 km∙h^−1^		15 km∙h^−1^		21 km∙h^−1^		CV% (9/15/21 km∙h^−1^)	MDC (9/15/21 km∙h^−1^)
		ICC	95%CI	ICC	95%CI	ICC	95%CI		
Spatiotemporal	CT (ms)	0.86	(0.70–0.95)	0.94	(0.84–0.97)	0.92	(0.79–0.97)	2.2/0.87/1.9	12.4/4.2/7.1
	ST (ms)	0.92	(0.81–0.97)	0.98	(0.94–0.99)	0.98	(0.94–0.99)	1.0/0.57/0.69	17.1/9.4/9.5
	SL (m)	0.92	(0.81–0.97)	0.98	(0.94–0.99)	0.98	(0.94–0.99)	1.0/0.57/0.69	0.04/0.04/0.06
	SF (strides∙min^−1^)	0.92	(0.81–0.97)	0.98	(0.95–0.99)	0.98	(0.94–0.99)	1.0/0.56/0.84	1.9/1.3/1.9
Kinetic	AP braking impulse (m∙s^−1^)	0.81	(0.55–0.92)	0.83	(0.57–0.93)	0.84	(0.62–0.94)	6.6/5.8/5.6	0.04/0.04/0.05
	AP prop. impulse (m∙s^−1^)	0.82	(0.58–0.93)	0.84	(0.63–0.94)	0.90	(0.75–0.96)	6.0/4.4/2.7	0.09/0.09/0.07
	VT peak brake (m∙s^−2^)	0.88	(0.71–0.95)	0.85	(0.65–0.94)	0.89	(0.73–0.96)	6.7/4.1/4.8	6.4/4.3/5.4

ST = Stride Time; SL = Stride Length; SF = Stride Frequency; CT = Contact Time; AP = Antero-Posterior; VT = Vertical. Correlations were all significant (p < 0.001)

## Discussion

The purpose of this study was to determine the test-retest reliability of some spatiotemporal and kinetic variables measured with the MTw IMU in a sample of endurance runners while running on a treadmill, and to analyse how this reliability changed with increasing speed. The main finding from this study was that the methodology used to estimate these variables from the MTw IMU can be considered reliable for measuring running biomechanics because good to excellent reliability was found (ICCs = 0.81–0.98). The spatiotemporal variables showed lower relative error (CV < 2.3%) than the kinetic variables (CV < 6.8%). For the whole group of variables, relative reliability was higher in the spatiotemporal variables, but the measurement error remained similar in both groups with increasing speeds.

### Spatiotemporal Variables

Average CT at 9 km∙h^−1^ was 241 ms and decreased to 201 ms and 161 ms at 15 km∙h^−1^ and at 21 km∙h^−1^, respectively (Table 1). A valid CT measurement is a key feature for IMU technology because the remaining of the spatiotemporal variables are calculated from CT. There is no consensus regarding the gold standard at which CT calculated with IMU should be validated. Instrumented treadmills might be the gold standard to validate IMU spatiotemporal measures (Sinclair et al., 2013; Watari et al., 2016), although most of the studies used other instrumentation such as overground platforms (Fadillioglu et al., 2020; Mo and Chow, 2018), photoelectrical systems (Garcia-Pinillos et al., 2021; Gindre et al., 2015), other validated accelerometers (Gouttebarge et al., 2015) or high-speed cameras (Chew et al., 2018; Garcia-Pinillos et al., 2019). To get an idea of the validity of the MTw IMU, we compared our results with those of other studies carried out with trained runners at different speeds in instrumented treadmills. For comparison purposes, we chose studies over the past decade in which the minimum vertical force used to define the stance was between 25 and 50 N of vertical force (Alcantara et al., 2021; Bredeweg et al., 2013; Giandolini et al., 2013; Girard et al., 2016; Hunter et al., 2019; Kluitenberg et al., 2012; Morin et al., 2012; 2019; Seeley et al., 2020; Weart et al., 2020). Figure 5 shows that our average CT values between 9 and 21 km∙h^−1^ are consistent with those measured with instrumented treadmills as reference standards.

A finding of this study is that the spatiotemporal variables were highly reliable with ICCs ranging from 0.86 to 0.98, and trivial CV values (< 2.3%) (Table 2). In agreement with Gouttebarge et al. (2015), the reliability was even higher at 15 and 21 km∙h^−1^ than at 9 km∙h^−1^. The lower reliability found at low speeds may be due to higher inter-subject variability because participants do not train or compete at such speeds. For a given speed, our reliability values compared favorably with those found in two studies carried out in recreational runners where the IMU was placed at the waist during overground running at speeds between 10 and 21 km∙h^−1^ (Gindre et al., 2015; Gouttebarge et al., 2015). However, it is difficult to compare the day-to-day reliability values between these studies because they markedly differed in several factors including the type and the placement of IMU, control of running speed, unknown formulas to calculate CT, and the running surface. For instance, in our study the treadmill speed was precisely controlled whereas less precision (up to 0.5 km∙h^−1^) was observed in the other studies. The higher speed control may explain, at least partially, our higher reliability values. Compared with reference instruments, only one study analyzed intraday reliability of CT while running on a treadmill at similar speeds, which found similar results to the ones of this study: good to excellent reliability (ICC = 0.79–0.96) and trivial CV values (< 3%) while running between 10 and 15 km∙h^−1^ on an instrumented treadmill (Van Alsenoy et al., 2018). It is therefore suggested that, due to the methodology used in this study, the spatiotemporal CT variable is highly reliable. Based on the MDC (Table 2), the smallest amount of change outside the error corresponded to ± 5% of the initial values at 9 km∙h^−1^ and to ± 4% at 21 km∙h^−1^.

### Kinetic Variables

In the AP axis, a typical running stance phase may be divided into two subcomponents (Hunter et al., 2005): 1) an initial braking phase, in which the force or acceleration direction opposes forward movement, and 2) a latter propulsion phase, in which the force or acceleration is consistent with the direction of forward motion. The impulse on the AP axis, defined as the area under the force-time curve, reflects the change in horizontal velocity of the CoM during the stance phase and has been related to sprint performance (Hunter et al., 2005; Kawamori et al., 2013).

To our knowledge, this is the first study using IMU technology trying to quantify accelerometry-based impulse variables while running on a treadmill at different speeds. The results show that increasing treadmill speed from 9 to 21 km•h^−1^ induced higher propulsive and braking impulses (Table 1): the magnitude of the increment with increased speed was much higher in the propulsive (63%) than in the braking impulse (21%) or the VT peak brake (17%). This pattern of change agrees with studies measuring overground reaction forces during submaximal (Munro et al., 1987) and sprint running (Hunter et al., 2005; Kawamori et al., 2013; Mero and Komi 1986; Morin et al., 2015), as well as running on an instrumented treadmill at different speeds (Girard et al., 2019). This suggests that the propulsive impulse rather than the breaking impulse or the VT peak is the major predictor of the change in the horizontal velocity during running.

The absolute values of the breaking impulse, quantified as a negative value to indicate its posterior direction, are in line with those reported in well-trained middle-distance runners running at seven speeds from 10 to 25 km•h^−1^ on an instrumented treadmill providing measurements of GRF (Girard et al., 2019). This is not the case with the absolute values of the propulsive impulses, because our values (from 0.47 m•s^−1^ at 9 km•h^−1^ to around 0.79 m•s^−1^ at 21 km•h^−1^, Table 1) are more than twice those found in the abovementioned study carried out with an instrumented treadmill. The reason for this discrepancy is unknown. Further research should be carried out to study the role of the AP negative acceleration (Figure 4A) during the aerial phase and its possible relevance to these higher propulsive impulse values during the contact phase measured by an IMU.

The relative reliability of the kinetic variables ranged from 0.81 to 0.90 and CV was small (< 6.8%) (Table 2). This is in line with studies measuring AP breaking and propulsive impulses from GRF during overground sprinting (Hunter et al., 2005; Kawamori et al., 2013). Based on the MDC of Table 2, the smallest amount of change outside the error must be higher than 15%, 11% and 10% at 9, 15, and 21 km∙h^−1^, respectively, to represent relevant clinical changes beyond the error of measurement.

Some limitations are present in this study: (1) the time-window used was considered sufficient to ensure the analysis of a minimum of strides in all participants. However, the higher average number of steps analysed at 21 km∙h^−1^ (~70) compared with 9 km∙h^−1^ (~54) could explain, at least partially, the better reliability found at higher speeds. (2) The present findings are likely to be only applicable to recreational endurance runners, at the range of standardised speeds and the surface area used. (3) Finally, there is also no consensus about the gold standard to be used to validate the IMU variables. Some authors suggest that IMUs should not be interchanged with the Newton unit of measurement, but could be considered as a standard on itself (Nedergaard et al., 2018; Raper et al., 2018). These limitations do not imply a detriment of the use of the methodology presented with the MTw IMU. The lower steps measured at lower speeds are deemed not to suppose low reliability. Compared to reference instruments, this lower-cost technology could be affordable for the type of the analysed sample environment, which often uses treadmills as a training method. Finally, beyond a validation process that requires appropriate reference instruments which often are not available, the acceleration patterns could become a standard which need further study to establish their role concerning both running performance and running-related injuries.

## Conclusions

The measurement error, expressed as relative (CV) and absolute values (MDC), allows to examine whether the variables experienced relevant changes beyond the error of measurement. These findings suggest that the methodology used with the MTw IMU is practically useful for monitoring individuals and ensuring real and correct changes over time. As a practical suggestion, runners who aim to increase their SF or decrease their braking impulses during running at 15 km∙h^−1^ measured by the MTw IMU, an increase greater than 1.3% in the SF and a minimum decrease of 13.5% in the braking impulse, will be required to ensure real changes based on the reliability of the sensor. This might be of particular interest for sport and exercise science practitioners, runners and coaches seeking reliable measures to quantify running mechanics. The similarity of the absolute values with studies which used reference instruments showed that these variables seem to be correctly estimated with the novel formulas from the CoM. Based on the results, the reliability of these spatiotemporal and kinetic variables measured with the MTw IMU in endurance runners while running on a treadmill was demonstrated. However, reliability varied depending on the speeds tested. Finally, the results of this study indicate the real change in spatiotemporal and kinetic variables to correctly interpret any improvement after an intervention period from IMU measurements.
